# 10-(4-Fluoro­phen­yl)-4-[(4-fluoro­phen­yl)amino]-5-phenyl-5,8,9,10-tetra­hydro­pyrimido[4,5-*b*]quin­olin-6(7*H*)-one

**DOI:** 10.1107/S2414314625007047

**Published:** 2025-08-12

**Authors:** Sizwe J. Zamisa, Adesola A. Adeleke, Bernard Omondi

**Affiliations:** a School of Agriculture and Science, Discipline of Chemistry, University of KwaZulu-Natal, Private Bag X54001, Durban, 4000, Republic of , South Africa; Katholieke Universiteit Leuven, Belgium

**Keywords:** crystal structure, pyrimidines

## Abstract

The asymmetric unit of the title compound, C_29_H_22_F_2_N_4_O, consists of one mol­ecule in which the pyrimidinyl and anilinyl units exhibit near coplanarity, subtending a dihedral angle of 10.22 (7)°. In contrast, the di­hydro­pyridine and phenyl rings are nearly perpendicular, making angles of 88.66 (7) and 89.14 (7)°. The crystal packing features alternating C—H⋯π and C—F⋯π inter­actions that generate a corrugated two-dimensional supra­molecular network in the crystallographic *ac* plane. This structure is further consolidated into a three-dimensional architecture by C—H⋯F hydrogen bonding.

## Abstract 2

The title compound forms a crystal structure with near-coplanar heteroaryl units and perpendicular aromatic rings, assembling into a corrugated two-dimensional network along the crystallographic *ac* plane that is consolidated into a three-dimensional supra­molecular architecture *via* hydrogen bonding.

## Graphical abstract

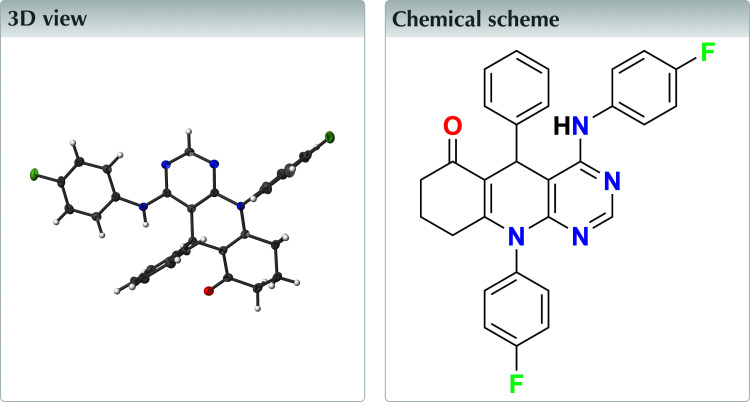

## Structure description

The title compound is a nitro­gen-rich heterocyclic mol­ecule belonging to the pyrimido[4,5-*b*]quinoline class, characterized by a fused pyrimidine ring and quinoline moiety. This class of compound is often synthesized using multicomponent reactions, allowing efficient isolation of target products through single-pot procedures (Moosavi-Zare & Najafi, 2023 ▸). Tetra­hydro­quinolines and their fused derivatives, such as pyrimidine, have gathered significant inter­est from pharmaceutical researchers due to their broad pharmacological properties, including anti­microbial, anti­cancer, anti­malarial, anti-inflammatory, and anti­histaminic activities (Patel *et al.*, 2024 ▸, Tawfeek *et al.*, 2024 ▸). Moreover, pyrimidine-containing motifs, apart from their notable biological activities, have served as inhibitors for Abelson kinase (AbI kinase) and protein tyrosine phosphatase 1B (PTP1B) in cell signalling as well as a DNA inter­calating agent (Esmaili *et al.*, 2022 ▸). As such, there is continuous inter­est from medicinal scientists in designing new pyrimidine-quinoline pharmacophore drugs with enhanced medicinal efficacy. In a continuation of our research inter­est (Zamisa *et al.*, 2023 ▸), we report herein the crystal structure of the title compound.

**Figure 1:**
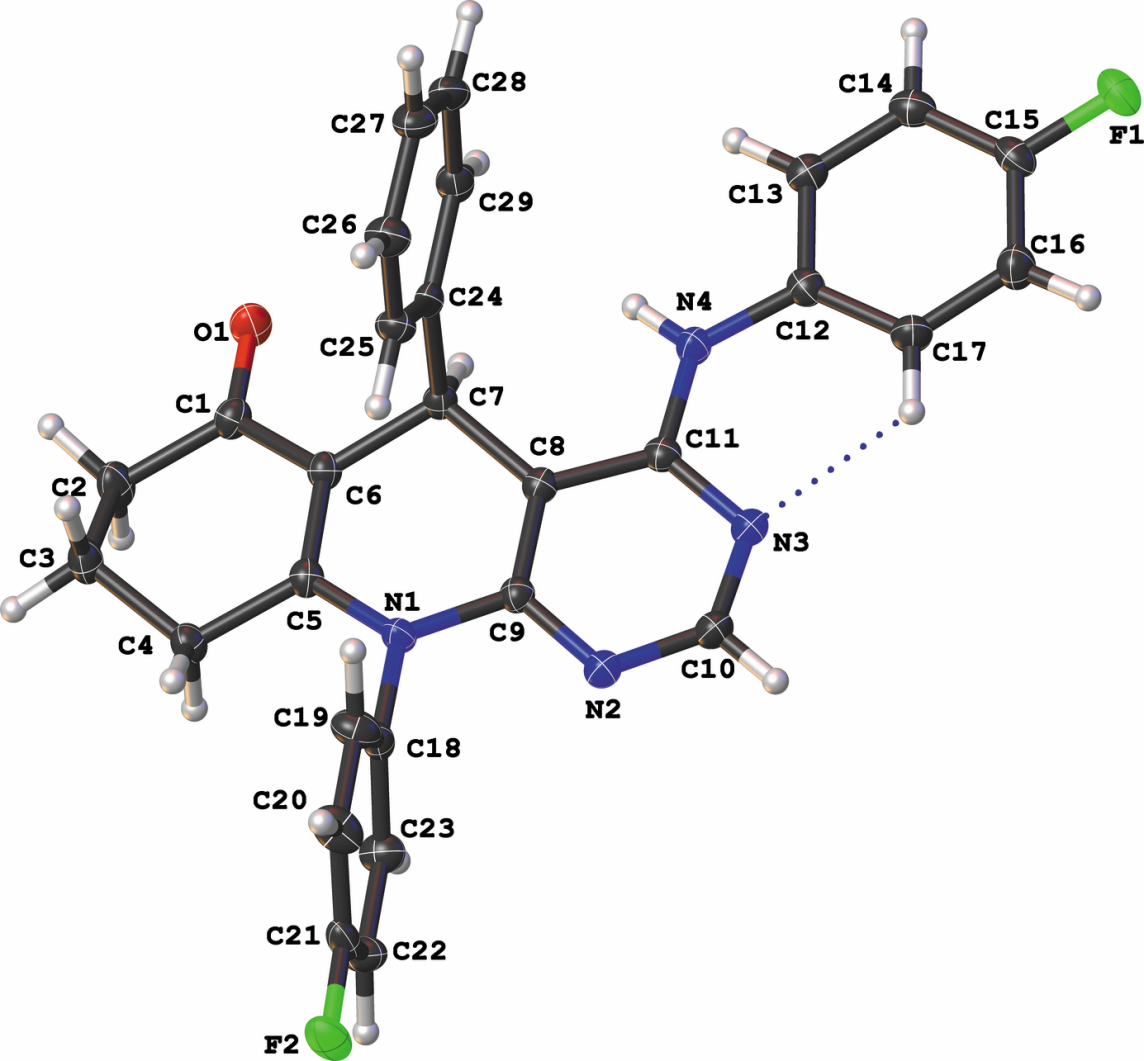
Mol­ecular structure of the title compound with displacement ellipsoids drawn at the 50% probability level.

**Figure 2:**
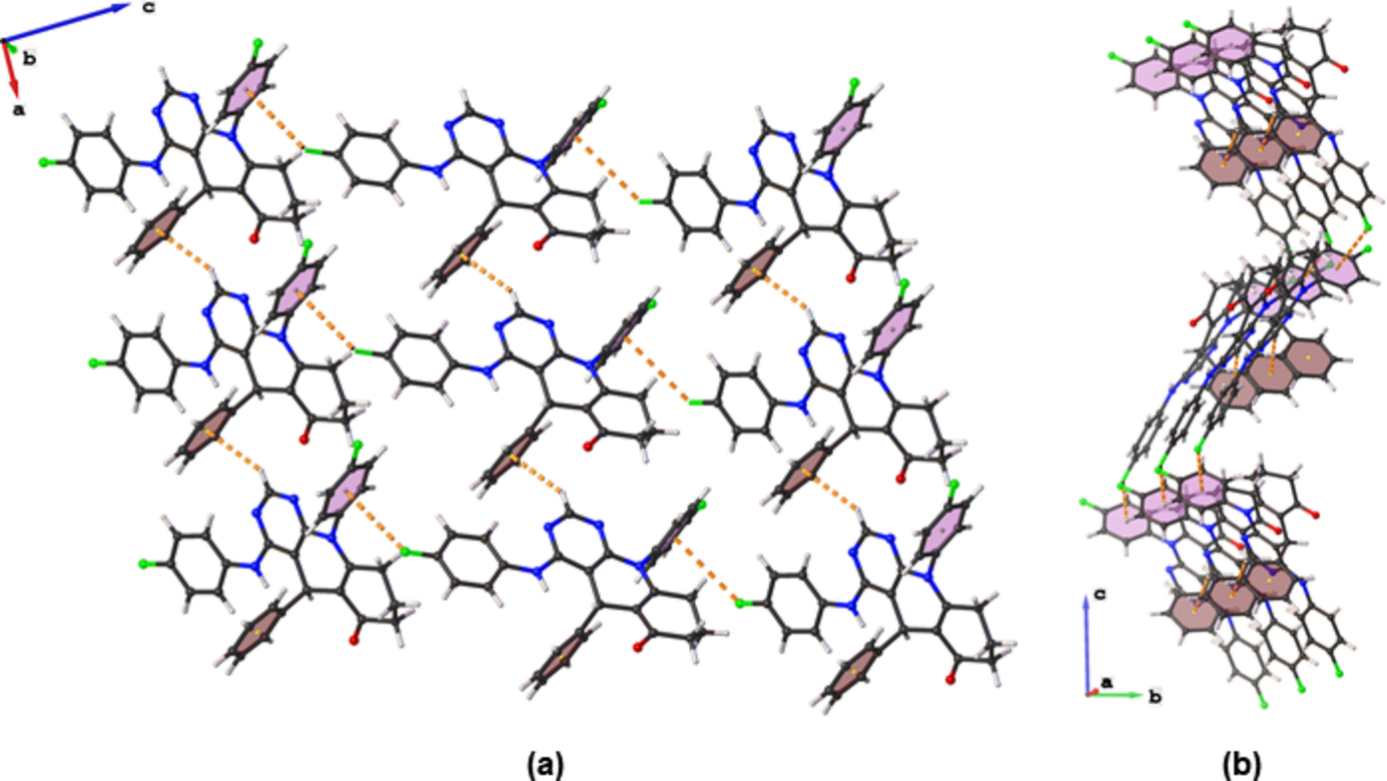
Representation of inter­molecular C10—H10⋯*Cg*1 and C15—F1⋯*Cg*2 inter­actions in the crystal packing of the title compound viewed with a slight rotation along the crystallographic (*a*) *b* and (*b*) *a* axes.

The asymmetric unit of the title compound contains one mol­ecule with a tetra­hydro­pyrimido[4,5-*b*]quinolin-6(7*H*)-one core, onto which the phenyl, 4-fluoro­phenyl and 4-fluoro­anilinyl moieties are attached on atoms C7, N1 and C11, respectively (Fig. 1 ▸). The dihedral angle between the pyrimidinyl and anilinyl moieties tends towards co-planarity [10.22 (7)°] while the dihedral angles between the central di­hydro­pyridine ring and the phenyl rings are 88.66 (7) and 89.14 (7)°. These values are comparable with those of reported chromeno­pyrimidine (Zamisa *et al.*, 2022 ▸) and hexa­hydro­quinolinyl formimidate (Zamisa & Omondi, 2022 ▸) derivatives. An intra­molecular C—H⋯N hydrogen bond occurs between atom H17 of the anilinyl ring and the N3 atom of the pyrimidine ring (Table 1 ▸). The crystal packing features alternating inter­molecular C10—H10⋯*Cg*1 and C15—F1⋯*Cg*2 inter­actions (Table 1 ▸), which form a corrugated two-dimensional supra­molecular structure that propagates in the crystallographic *ac* plane as depicted in Fig. 2 ▸. These corrugated supra­molecular sheets are further linked by C3—H3*A*⋯F2 and C4—H4*A*⋯F1 hydrogen bonds (Table 1 ▸), resulting in a three-dimensional supra­molecular architecture.

## Synthesis and crystallization

The precursors, 2-amino-1-(4-fluoro­phen­yl)-5-oxo-4-phenyl-1,4,5,6,7,8-hexa­hydro­quinoline-3-carbo­nitrile and ethyl (*E*)-*N*-[3-cyano-1-(4-fluoro­phen­yl)-5-oxo-4-phenyl-1,4,5,6,7,8-hexa­hydro­quinolin-2-yl]formimidate were synthesized using modified literature procedures (Zamisa *et al.*, 2022 ▸; Zamisa & Omondi, 2022 ▸). The title compound was synthesized by the following procedure. A solution of ethyl (*E*)-*N*-[3-cyano-1-(4-fluoro­phen­yl)-5-oxo-4-phenyl-1,4,5,6,7,8-hexa­hydro­naphtha­len-2-yl]formimidate (1 mmol) and the corresponding 4-fluoro­aniline (1.2 mmol) in 10 ml of acetic acid was placed into a sealed 30 ml pressurized vial. The reaction mixture was exposed to microwave irradiation at 200 W using a single-mode microwave synthesis system, with the temperature maintained at 413 K for 20 minutes. The formation of the product was confirmed using thin-layer chromatography (TLC). Upon completion, distilled water was carefully layered onto the reaction mixture without agitation, resulting in the formation of a turbid suspension. This was allowed to stand overnight. The precipitated crude product was collected by vacuum filtration, washed with distilled water, and subsequently purified by recrystallization from a mixed solvent system of ethanol and water (Zamisa *et al.*, 2023 ▸).

## Refinement

Crystal data, data collection and structure refinement details are summarized in Table 2 ▸.

## Supplementary Material

Crystal structure: contains datablock(s) I. DOI: 10.1107/S2414314625007047/vm4070sup1.cif

Structure factors: contains datablock(s) I. DOI: 10.1107/S2414314625007047/vm4070Isup2.hkl

Supporting information file. DOI: 10.1107/S2414314625007047/vm4070Isup3.cml

CCDC reference: 2478466

Additional supporting information:  crystallographic information; 3D view; checkCIF report

## Figures and Tables

**Table 1:** Hydrogen-bond geometry (Å, °) *Cg*1 and *Cg*2 are centroids of the C24–C29 and C18–C23 rings, respectively.

*D*—H⋯*A*	*D*—H	H⋯*A*	*D*⋯*A*	*D*—H⋯*A*
C3—H3*A*⋯F2^i^	0.99	2.70	3.3379 (18)	123
C4—H4*A*⋯F1^ii^	0.99	2.54	3.3829 (18)	143
C17—H17⋯N3	0.95	2.23	2.856 (2)	123
C10—H10⋯*Cg*1^iii^	0.95	2.71	3.5471 (17)	147
C15—F1⋯*Cg*2^iv^	1.3673 (18)	3.8721 (12)	4.7637 (18)	123.40 (9)

Symmetry codes: (i) 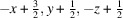; (ii) 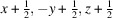; (iii) 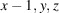; (iv) 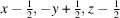.

**Table 2:** Experimental details

Crystal data	
Chemical formula	C_29_H_22_F_2_N_4_O
*M*_r_	480.50
Crystal system, space group	Monoclinic, *P*2_1_/*n*
Temperature (K)	100
*a*, *b*, *c* (Å)	8.4575 (4), 11.5163 (5), 23.0761 (10)
β (°)	91.103 (2)
*V* (Å^3^)	2247.17 (17)
*Z*	4
Radiation type	Mo *K*α
μ (mm^−1^)	0.10
Crystal size (mm)	0.22 × 0.14 × 0.13

Data collection	
Diffractometer	Bruker *SMART* APEXII area detector
Absorption correction	Multi-scan (*SADABS*; Krause *et al.*, 2015 ▸)
*T*_min_, *T*_max_	0.688, 0.746
No. of measured, independent and observed [*I* > 2σ(*I*)] reflections	15704, 5044, 3821
*R*_int_	0.030
(sin θ/λ)_max_ (Å^−1^)	0.650

Refinement	
*R*[*F*^2^ > 2σ(*F*^2^)], *wR*(*F*^2^), *S*	0.042, 0.113, 1.04
No. of reflections	5044
No. of parameters	325
H-atom treatment	H-atom parameters constrained
Δρ_max_, Δρ_min_ (e Å^−3^)	0.30, −0.24

Computer programs: *APEX2* and *SAINT* (Bruker, 2014 ▸), *SHELXT* (Sheldrick, 2015*a* ▸), *SHELXL2018/3* (Sheldrick, 2015*b* ▸) and *OLEX2* (Dolomanov *et al.*, 2009 ▸).

## full crystallographic data

### 10-(4-Fluorophenyl)-4-[(4-fluorophenyl)amino]-5-phenyl-5,8,9,10-tetrahydropyrimido[4,5-b]quinolin-6(7H)-one Crystal data

**Table:**

C_29_H_22_F_2_N_4_O	*F*(000) = 1000
*M**_r_* = 480.50	*D*_x_ = 1.420 Mg m^−^^3^
Monoclinic, *P*2_1_/*n*	Mo *K*α radiation, λ = 0.71073 Å
*a* = 8.4575 (4) Å	Cell parameters from 4354 reflections
*b* = 11.5163 (5) Å	θ = 2.6–27.1°
*c* = 23.0761 (10) Å	µ = 0.10 mm^−^^1^
β = 91.103 (2)°	*T* = 100 K
*V* = 2247.17 (17) Å^3^	Block, colourless
*Z* = 4	0.22 × 0.14 × 0.13 mm

### 10-(4-Fluorophenyl)-4-[(4-fluorophenyl)amino]-5-phenyl-5,8,9,10-tetrahydropyrimido[4,5-b]quinolin-6(7H)-one Data collection

**Table:**

Bruker SMART APEXII area detector diffractometer	3821 reflections with *I* > 2σ(*I*)
Detector resolution: 7.9 pixels mm^-1^	*R*_int_ = 0.030
ω and φ scans	θ_max_ = 27.5°, θ_min_ = 1.8°
Absorption correction: multi-scan (SADABS; Krause *et al.*, 2015)	*h* = −10→10
*T*_min_ = 0.688, *T*_max_ = 0.746	*k* = −14→12
15704 measured reflections	*l* = −27→29
5044 independent reflections	

### 10-(4-Fluorophenyl)-4-[(4-fluorophenyl)amino]-5-phenyl-5,8,9,10-tetrahydropyrimido[4,5-b]quinolin-6(7H)-one Refinement

**Table:**

Refinement on *F*^2^	0 restraints
Least-squares matrix: full	Hydrogen site location: inferred from neighbouring sites
*R*[*F*^2^ > 2σ(*F*^2^)] = 0.042	H-atom parameters constrained
*wR*(*F*^2^) = 0.113	*w* = 1/[σ^2^(*F*_o_^2^) + (0.0529*P*)^2^ + 0.6852*P*] where *P* = (*F*_o_^2^ + 2*F*_c_^2^)/3
*S* = 1.04	(Δ/σ)_max_ = 0.001
5044 reflections	Δρ_max_ = 0.30 e Å^−^^3^
325 parameters	Δρ_min_ = −0.24 e Å^−^^3^

### 10-(4-Fluorophenyl)-4-[(4-fluorophenyl)amino]-5-phenyl-5,8,9,10-tetrahydropyrimido[4,5-b]quinolin-6(7H)-one Special details

**Table:**

Geometry. All esds (except the esd in the dihedral angle between two l.s. planes) are estimated using the full covariance matrix. The cell esds are taken into account individually in the estimation of esds in distances, angles and torsion angles; correlations between esds in cell parameters are only used when they are defined by crystal symmetry. An approximate (isotropic) treatment of cell esds is used for estimating esds involving l.s. planes.

### 10-(4-Fluorophenyl)-4-[(4-fluorophenyl)amino]-5-phenyl-5,8,9,10-tetrahydropyrimido[4,5-b]quinolin-6(7H)-one Fractional atomic coordinates and isotropic or equivalent isotropic displacement parameters (Å^2^)

**Table:**

	*x*	*y*	*z*	*U*_iso_*/*U*_eq_	
F1	0.33017 (12)	0.62197 (8)	−0.25793 (4)	0.0297 (2)	
F2	0.29340 (12)	−0.21604 (8)	0.21262 (4)	0.0294 (2)	
O1	1.03263 (13)	0.39741 (9)	0.08347 (5)	0.0245 (3)	
N1	0.59222 (15)	0.14969 (11)	0.10474 (5)	0.0179 (3)	
N2	0.35335 (15)	0.17807 (11)	0.05491 (6)	0.0218 (3)	
N3	0.33012 (15)	0.31331 (12)	−0.02316 (6)	0.0227 (3)	
N4	0.54889 (15)	0.41642 (11)	−0.05497 (6)	0.0198 (3)	
H4	0.645825	0.438667	−0.045957	0.024*	
C1	0.96609 (18)	0.32095 (13)	0.11089 (7)	0.0199 (3)	
C2	1.0312 (2)	0.28246 (14)	0.16912 (7)	0.0253 (4)	
H2A	0.991420	0.335061	0.199479	0.030*	
H2B	1.147946	0.288722	0.169206	0.030*	
C3	0.98538 (19)	0.15879 (14)	0.18359 (7)	0.0238 (4)	
H3A	1.020579	0.140501	0.223727	0.029*	
H3B	1.039324	0.104692	0.157122	0.029*	
C4	0.80745 (18)	0.14177 (13)	0.17790 (7)	0.0205 (3)	
H4A	0.782934	0.057900	0.180538	0.025*	
H4B	0.755423	0.181460	0.210470	0.025*	
C5	0.74171 (18)	0.18859 (12)	0.12155 (6)	0.0174 (3)	
C6	0.81959 (17)	0.26783 (13)	0.08921 (6)	0.0175 (3)	
C7	0.75833 (17)	0.30856 (12)	0.03066 (6)	0.0166 (3)	
H7	0.777238	0.394037	0.027622	0.020*	
C8	0.58194 (17)	0.28690 (12)	0.02576 (6)	0.0167 (3)	
C9	0.50765 (17)	0.20664 (12)	0.06025 (7)	0.0174 (3)	
C10	0.27491 (19)	0.23402 (14)	0.01319 (7)	0.0240 (4)	
H10	0.165990	0.215220	0.008707	0.029*	
C11	0.48470 (18)	0.33891 (13)	−0.01736 (6)	0.0175 (3)	
C12	0.48445 (18)	0.46671 (13)	−0.10615 (7)	0.0194 (3)	
C13	0.58433 (19)	0.54076 (13)	−0.13647 (7)	0.0216 (3)	
H13	0.688356	0.555536	−0.122051	0.026*	
C14	0.53219 (19)	0.59258 (13)	−0.18741 (7)	0.0227 (3)	
H14	0.599581	0.643217	−0.208067	0.027*	
C15	0.3814 (2)	0.56981 (13)	−0.20777 (7)	0.0223 (3)	
C16	0.2817 (2)	0.49701 (14)	−0.17937 (7)	0.0250 (4)	
H16	0.178784	0.481759	−0.194753	0.030*	
C17	0.33191 (19)	0.44550 (14)	−0.12784 (7)	0.0231 (3)	
H17	0.262662	0.395920	−0.107386	0.028*	
C18	0.51627 (17)	0.05440 (13)	0.13387 (7)	0.0182 (3)	
C19	0.5343 (2)	−0.05689 (14)	0.11286 (7)	0.0254 (4)	
H19	0.597466	−0.070338	0.079930	0.030*	
C20	0.4600 (2)	−0.14929 (14)	0.13987 (7)	0.0280 (4)	
H20	0.472089	−0.226446	0.126080	0.034*	
C21	0.36898 (19)	−0.12621 (13)	0.18683 (7)	0.0218 (3)	
C22	0.3508 (2)	−0.01677 (14)	0.20913 (7)	0.0255 (4)	
H22	0.288699	−0.003995	0.242402	0.031*	
C23	0.42544 (19)	0.07463 (14)	0.18186 (7)	0.0239 (4)	
H23	0.414073	0.151428	0.196229	0.029*	
C24	0.84253 (17)	0.24862 (13)	−0.01892 (6)	0.0172 (3)	
C25	0.84009 (19)	0.12777 (13)	−0.02272 (7)	0.0208 (3)	
H25	0.788372	0.083846	0.006156	0.025*	
C26	0.9120 (2)	0.07107 (14)	−0.06799 (7)	0.0248 (4)	
H26	0.910975	−0.011333	−0.069820	0.030*	
C27	0.9855 (2)	0.13496 (15)	−0.11071 (7)	0.0262 (4)	
H27	1.034550	0.096452	−0.142030	0.031*	
C28	0.98724 (19)	0.25435 (15)	−0.10761 (7)	0.0250 (4)	
H28	1.036136	0.298015	−0.137247	0.030*	
C29	0.91813 (18)	0.31154 (14)	−0.06152 (7)	0.0216 (3)	
H29	0.922649	0.393837	−0.059185	0.026*	

### 10-(4-Fluorophenyl)-4-[(4-fluorophenyl)amino]-5-phenyl-5,8,9,10-tetrahydropyrimido[4,5-b]quinolin-6(7H)-one Atomic displacement parameters (Å^2^)

**Table:**

	*U*^11^	*U*^22^	*U*^33^	*U*^12^	*U*^13^	*U*^23^
F1	0.0376 (6)	0.0307 (5)	0.0208 (5)	0.0030 (4)	−0.0025 (4)	0.0053 (4)
F2	0.0345 (6)	0.0263 (5)	0.0273 (5)	−0.0106 (4)	−0.0033 (4)	0.0097 (4)
O1	0.0230 (6)	0.0226 (6)	0.0279 (6)	−0.0064 (5)	−0.0030 (5)	0.0029 (5)
N1	0.0176 (6)	0.0190 (6)	0.0171 (7)	−0.0022 (5)	−0.0007 (5)	0.0033 (5)
N2	0.0176 (7)	0.0252 (7)	0.0227 (7)	−0.0010 (5)	0.0002 (5)	0.0031 (6)
N3	0.0168 (7)	0.0278 (7)	0.0235 (7)	0.0004 (5)	0.0009 (5)	0.0044 (6)
N4	0.0166 (6)	0.0215 (6)	0.0213 (7)	−0.0019 (5)	−0.0019 (5)	0.0025 (5)
C1	0.0183 (8)	0.0180 (7)	0.0232 (8)	−0.0001 (6)	−0.0013 (6)	−0.0019 (6)
C2	0.0247 (8)	0.0261 (8)	0.0248 (9)	−0.0059 (7)	−0.0081 (7)	0.0019 (7)
C3	0.0232 (8)	0.0245 (8)	0.0234 (9)	−0.0013 (6)	−0.0068 (6)	0.0026 (7)
C4	0.0221 (8)	0.0213 (8)	0.0180 (8)	−0.0006 (6)	−0.0025 (6)	0.0005 (6)
C5	0.0183 (7)	0.0162 (7)	0.0176 (8)	0.0013 (6)	−0.0017 (6)	−0.0026 (6)
C6	0.0177 (7)	0.0165 (7)	0.0180 (8)	0.0002 (6)	−0.0017 (6)	−0.0011 (6)
C7	0.0161 (7)	0.0149 (7)	0.0189 (8)	−0.0020 (5)	−0.0011 (6)	0.0011 (6)
C8	0.0170 (7)	0.0161 (7)	0.0171 (7)	0.0006 (5)	0.0002 (6)	−0.0022 (6)
C9	0.0171 (7)	0.0180 (7)	0.0172 (8)	0.0018 (6)	0.0002 (6)	−0.0012 (6)
C10	0.0170 (8)	0.0294 (9)	0.0258 (9)	−0.0003 (6)	0.0015 (6)	0.0044 (7)
C11	0.0187 (7)	0.0170 (7)	0.0170 (8)	0.0013 (6)	0.0024 (6)	−0.0018 (6)
C12	0.0214 (8)	0.0177 (7)	0.0191 (8)	0.0025 (6)	0.0007 (6)	−0.0009 (6)
C13	0.0201 (8)	0.0214 (8)	0.0234 (9)	−0.0009 (6)	0.0018 (6)	−0.0019 (6)
C14	0.0271 (9)	0.0186 (8)	0.0226 (9)	0.0000 (6)	0.0063 (7)	−0.0008 (6)
C15	0.0312 (9)	0.0200 (8)	0.0158 (8)	0.0042 (6)	0.0016 (6)	0.0007 (6)
C16	0.0231 (8)	0.0266 (8)	0.0252 (9)	−0.0004 (7)	−0.0035 (7)	0.0013 (7)
C17	0.0211 (8)	0.0239 (8)	0.0244 (9)	−0.0029 (6)	0.0020 (6)	0.0040 (7)
C18	0.0173 (7)	0.0190 (7)	0.0181 (8)	−0.0009 (6)	−0.0031 (6)	0.0028 (6)
C19	0.0336 (9)	0.0234 (8)	0.0195 (8)	−0.0020 (7)	0.0053 (7)	−0.0019 (7)
C20	0.0423 (10)	0.0183 (8)	0.0234 (9)	−0.0031 (7)	−0.0006 (8)	−0.0029 (7)
C21	0.0223 (8)	0.0226 (8)	0.0205 (8)	−0.0050 (6)	−0.0053 (6)	0.0076 (6)
C22	0.0247 (8)	0.0262 (8)	0.0258 (9)	0.0027 (7)	0.0080 (7)	0.0044 (7)
C23	0.0262 (8)	0.0182 (7)	0.0274 (9)	0.0021 (6)	0.0048 (7)	0.0000 (7)
C24	0.0124 (7)	0.0216 (7)	0.0174 (8)	−0.0011 (6)	−0.0033 (6)	0.0019 (6)
C25	0.0210 (8)	0.0213 (8)	0.0200 (8)	−0.0011 (6)	0.0034 (6)	0.0033 (6)
C26	0.0277 (9)	0.0216 (8)	0.0253 (9)	0.0014 (6)	0.0043 (7)	0.0000 (7)
C27	0.0237 (8)	0.0335 (9)	0.0215 (8)	0.0005 (7)	0.0046 (7)	−0.0018 (7)
C28	0.0198 (8)	0.0352 (9)	0.0201 (8)	−0.0066 (7)	0.0014 (6)	0.0055 (7)
C29	0.0203 (8)	0.0201 (8)	0.0244 (9)	−0.0040 (6)	−0.0024 (6)	0.0041 (6)

### 10-(4-Fluorophenyl)-4-[(4-fluorophenyl)amino]-5-phenyl-5,8,9,10-tetrahydropyrimido[4,5-b]quinolin-6(7H)-one Geometric parameters (Å, º)

**Table:**

F1—C15	1.3673 (18)	C12—C13	1.398 (2)
F2—C21	1.3593 (17)	C12—C17	1.396 (2)
O1—C1	1.2272 (19)	C13—H13	0.9500
N1—C5	1.3896 (19)	C13—C14	1.383 (2)
N1—C9	1.4027 (19)	C14—H14	0.9500
N1—C18	1.4441 (19)	C14—C15	1.375 (2)
N2—C9	1.3492 (19)	C15—C16	1.366 (2)
N2—C10	1.326 (2)	C16—H16	0.9500
N3—C10	1.331 (2)	C16—C17	1.388 (2)
N3—C11	1.344 (2)	C17—H17	0.9500
N4—H4	0.8800	C18—C19	1.380 (2)
N4—C11	1.3649 (19)	C18—C23	1.380 (2)
N4—C12	1.4151 (19)	C19—H19	0.9500
C1—C2	1.509 (2)	C19—C20	1.390 (2)
C1—C6	1.462 (2)	C20—H20	0.9500
C2—H2A	0.9900	C20—C21	1.367 (2)
C2—H2B	0.9900	C21—C22	1.371 (2)
C2—C3	1.515 (2)	C22—H22	0.9500
C3—H3A	0.9900	C22—C23	1.385 (2)
C3—H3B	0.9900	C23—H23	0.9500
C3—C4	1.521 (2)	C24—C25	1.395 (2)
C4—H4A	0.9900	C24—C29	1.387 (2)
C4—H4B	0.9900	C25—H25	0.9500
C4—C5	1.504 (2)	C25—C26	1.382 (2)
C5—C6	1.357 (2)	C26—H26	0.9500
C6—C7	1.512 (2)	C26—C27	1.387 (2)
C7—H7	1.0000	C27—H27	0.9500
C7—C8	1.515 (2)	C27—C28	1.377 (2)
C7—C24	1.524 (2)	C28—H28	0.9500
C8—C9	1.379 (2)	C28—C29	1.389 (2)
C8—C11	1.412 (2)	C29—H29	0.9500
C10—H10	0.9500		
			
C5—N1—C9	120.14 (12)	C17—C12—N4	124.47 (14)
C5—N1—C18	121.70 (12)	C17—C12—C13	119.37 (14)
C9—N1—C18	118.10 (12)	C12—C13—H13	119.9
C10—N2—C9	114.67 (13)	C14—C13—C12	120.20 (15)
C10—N3—C11	116.11 (13)	C14—C13—H13	119.9
C11—N4—H4	114.9	C13—C14—H14	120.5
C11—N4—C12	130.30 (13)	C15—C14—C13	119.10 (15)
C12—N4—H4	114.9	C15—C14—H14	120.5
O1—C1—C2	120.45 (14)	F1—C15—C14	118.83 (14)
O1—C1—C6	121.20 (14)	C16—C15—F1	119.15 (15)
C6—C1—C2	118.32 (13)	C16—C15—C14	122.02 (15)
C1—C2—H2A	109.1	C15—C16—H16	120.3
C1—C2—H2B	109.1	C15—C16—C17	119.45 (15)
C1—C2—C3	112.46 (13)	C17—C16—H16	120.3
H2A—C2—H2B	107.8	C12—C17—H17	120.1
C3—C2—H2A	109.1	C16—C17—C12	119.85 (15)
C3—C2—H2B	109.1	C16—C17—H17	120.1
C2—C3—H3A	109.4	C19—C18—N1	119.29 (14)
C2—C3—H3B	109.4	C23—C18—N1	120.32 (13)
C2—C3—C4	111.04 (13)	C23—C18—C19	120.39 (15)
H3A—C3—H3B	108.0	C18—C19—H19	120.0
C4—C3—H3A	109.4	C18—C19—C20	119.98 (15)
C4—C3—H3B	109.4	C20—C19—H19	120.0
C3—C4—H4A	109.2	C19—C20—H20	120.9
C3—C4—H4B	109.2	C21—C20—C19	118.23 (15)
H4A—C4—H4B	107.9	C21—C20—H20	120.9
C5—C4—C3	112.16 (13)	F2—C21—C20	118.41 (14)
C5—C4—H4A	109.2	F2—C21—C22	118.57 (15)
C5—C4—H4B	109.2	C20—C21—C22	123.03 (15)
N1—C5—C4	116.42 (13)	C21—C22—H22	120.9
C6—C5—N1	120.77 (13)	C21—C22—C23	118.22 (15)
C6—C5—C4	122.76 (14)	C23—C22—H22	120.9
C1—C6—C7	116.72 (13)	C18—C23—C22	120.14 (15)
C5—C6—C1	120.68 (14)	C18—C23—H23	119.9
C5—C6—C7	122.57 (13)	C22—C23—H23	119.9
C6—C7—H7	108.4	C25—C24—C7	119.51 (13)
C6—C7—C8	109.61 (12)	C29—C24—C7	121.58 (13)
C6—C7—C24	111.91 (12)	C29—C24—C25	118.89 (14)
C8—C7—H7	108.4	C24—C25—H25	119.6
C8—C7—C24	110.07 (12)	C26—C25—C24	120.84 (15)
C24—C7—H7	108.4	C26—C25—H25	119.6
C9—C8—C7	121.76 (13)	C25—C26—H26	120.1
C9—C8—C11	115.17 (13)	C25—C26—C27	119.75 (15)
C11—C8—C7	122.83 (13)	C27—C26—H26	120.1
N2—C9—N1	115.56 (13)	C26—C27—H27	120.1
N2—C9—C8	124.16 (14)	C28—C27—C26	119.81 (16)
C8—C9—N1	120.28 (13)	C28—C27—H27	120.1
N2—C10—N3	127.92 (15)	C27—C28—H28	119.7
N2—C10—H10	116.0	C27—C28—C29	120.60 (15)
N3—C10—H10	116.0	C29—C28—H28	119.7
N3—C11—N4	118.57 (13)	C24—C29—C28	120.08 (15)
N3—C11—C8	121.95 (14)	C24—C29—H29	120.0
N4—C11—C8	119.48 (13)	C28—C29—H29	120.0
C13—C12—N4	116.15 (14)		
			
F1—C15—C16—C17	−178.78 (14)	C9—N1—C18—C19	90.37 (17)
F2—C21—C22—C23	−178.63 (14)	C9—N1—C18—C23	−89.01 (18)
O1—C1—C2—C3	−154.05 (15)	C9—N2—C10—N3	0.0 (2)
O1—C1—C6—C5	−175.83 (14)	C9—C8—C11—N3	1.4 (2)
O1—C1—C6—C7	2.2 (2)	C9—C8—C11—N4	−177.69 (13)
N1—C5—C6—C1	171.24 (13)	C10—N2—C9—N1	179.06 (13)
N1—C5—C6—C7	−6.7 (2)	C10—N2—C9—C8	−0.2 (2)
N1—C18—C19—C20	−179.02 (15)	C10—N3—C11—N4	177.55 (14)
N1—C18—C23—C22	179.00 (14)	C10—N3—C11—C8	−1.6 (2)
N4—C12—C13—C14	179.10 (14)	C11—N3—C10—N2	0.8 (3)
N4—C12—C17—C16	−178.27 (15)	C11—N4—C12—C13	−177.63 (14)
C1—C2—C3—C4	−52.94 (19)	C11—N4—C12—C17	1.3 (3)
C1—C6—C7—C8	−156.38 (13)	C11—C8—C9—N1	−179.72 (13)
C1—C6—C7—C24	81.22 (16)	C11—C8—C9—N2	−0.5 (2)
C2—C1—C6—C5	2.0 (2)	C12—N4—C11—N3	−9.4 (2)
C2—C1—C6—C7	−179.90 (13)	C12—N4—C11—C8	169.70 (14)
C2—C3—C4—C5	48.65 (18)	C12—C13—C14—C15	−0.3 (2)
C3—C4—C5—N1	162.59 (13)	C13—C12—C17—C16	0.6 (2)
C3—C4—C5—C6	−19.8 (2)	C13—C14—C15—F1	179.52 (13)
C4—C5—C6—C1	−6.3 (2)	C13—C14—C15—C16	−0.3 (2)
C4—C5—C6—C7	175.74 (14)	C14—C15—C16—C17	1.1 (2)
C5—N1—C9—N2	−167.20 (13)	C15—C16—C17—C12	−1.2 (2)
C5—N1—C9—C8	12.1 (2)	C17—C12—C13—C14	0.1 (2)
C5—N1—C18—C19	−92.66 (18)	C18—N1—C5—C4	−10.9 (2)
C5—N1—C18—C23	87.96 (18)	C18—N1—C5—C6	171.44 (14)
C5—C6—C7—C8	21.64 (19)	C18—N1—C9—N2	9.82 (19)
C5—C6—C7—C24	−100.77 (16)	C18—N1—C9—C8	−170.89 (13)
C6—C1—C2—C3	28.0 (2)	C18—C19—C20—C21	0.6 (3)
C6—C7—C8—C9	−20.93 (19)	C19—C18—C23—C22	−0.4 (2)
C6—C7—C8—C11	164.88 (13)	C19—C20—C21—F2	178.62 (14)
C6—C7—C24—C25	57.60 (18)	C19—C20—C21—C22	−1.5 (3)
C6—C7—C24—C29	−124.27 (15)	C20—C21—C22—C23	1.5 (3)
C7—C8—C9—N1	5.7 (2)	C21—C22—C23—C18	−0.5 (2)
C7—C8—C9—N2	−175.10 (14)	C23—C18—C19—C20	0.4 (2)
C7—C8—C11—N3	175.97 (14)	C24—C7—C8—C9	102.56 (16)
C7—C8—C11—N4	−3.1 (2)	C24—C7—C8—C11	−71.63 (17)
C7—C24—C25—C26	178.36 (14)	C24—C25—C26—C27	−1.0 (2)
C7—C24—C29—C28	−176.93 (14)	C25—C24—C29—C28	1.2 (2)
C8—C7—C24—C25	−64.54 (17)	C25—C26—C27—C28	0.4 (2)
C8—C7—C24—C29	113.58 (15)	C26—C27—C28—C29	1.0 (2)
C9—N1—C5—C4	166.05 (13)	C27—C28—C29—C24	−1.8 (2)
C9—N1—C5—C6	−11.7 (2)	C29—C24—C25—C26	0.2 (2)

### 10-(4-Fluorophenyl)-4-[(4-fluorophenyl)amino]-5-phenyl-5,8,9,10-tetrahydropyrimido[4,5-b]quinolin-6(7H)-one Hydrogen-bond geometry (Å, º)

*Cg*1 and *Cg*2 are centroids of the C24–C29 and C18–C23 rings, respectively.

**Table:**

*D*—H···*A*	*D*—H	H···*A*	*D*···*A*	*D*—H···*A*
C3—H3*A*···F2^i^	0.99	2.70	3.3379 (18)	123
C4—H4*A*···F1^ii^	0.99	2.54	3.3829 (18)	143
C17—H17···N3	0.95	2.23	2.856 (2)	123
C10—H10···*Cg*1^iii^	0.95	2.71	3.5471 (17)	147
C15—F1···*Cg*2^iv^	1.37 (1)	3.87 (1)	4.7637 (18)	123 (1)

Symmetry codes: (i) −*x*+3/2, *y*+1/2, −*z*+1/2; (ii) *x*+1/2, −*y*+1/2, *z*+1/2; (iii) *x*−1, *y*, *z*; (iv) *x*−1/2, −*y*+1/2, *z*−1/2.
